# Remission of Ulcerated Necrobiosis Lipoidica Diabeticorum after Bariatric Surgery

**DOI:** 10.1155/2013/352579

**Published:** 2013-05-16

**Authors:** Suleyman Bozkurt, Halil Coskun, Huseyin Kadioglu, Naim Memmi, Gokhan Cipe, Yeliz Emine Ersoy, Banu Lebe, Mahmut Muslumanoglu

**Affiliations:** ^1^Department of Surgery, Faculty of Medicine, Bezmialem Vakif University, Adnan Menderes Bulvari, Vatan Caddesi, Fatih, 34093 Istanbul, Turkey; ^2^Department of Pathology, Faculty of Medicine, Dokuz Eylul University, 35340 Izmir, Turkey

## Abstract

A 32-year-old woman with type 2 diabetes mellitus suffering from morbid obesity with BMI 45,14 kg/m^2^ was operated on. Not only the type 2DM but also one of its complication known as necrobiosis lipoidica diabeticorum remitted postoperatively. Obesity should no longer be regarded simply as a cosmetic problem affecting certain individuals but an epidemic that threatens global well-being. It causes or exacerbates many health problems, and in particular, it is associated with the type 2 diabetes. Necrobiosis lipoidica is a granulomatous skin disease of unknown etiology, associated mainly with diabetes mellitus. We presented in this paper a morbid obese case of necrobiosis lipoidica diabeticorum with dramatic good response to bariatric surgery.

## 1. Introduction

Obesity should no longer be regarded simply as a cosmetic problem affecting certain individuals but an epidemic that threatens global well-being. It causes or exacerbates many health problems, and in particular, it is associated with the T2DM.

Necrobiosis lipoidica is a granulomatous skin disease of unknown etiology, associated with diabetes mellitus which responds with limited success to many treatment options. We report in this paper a case of NLD treated successfully with bariatric/metabolic surgery.

## 2. Case Report

**Figure 1:**
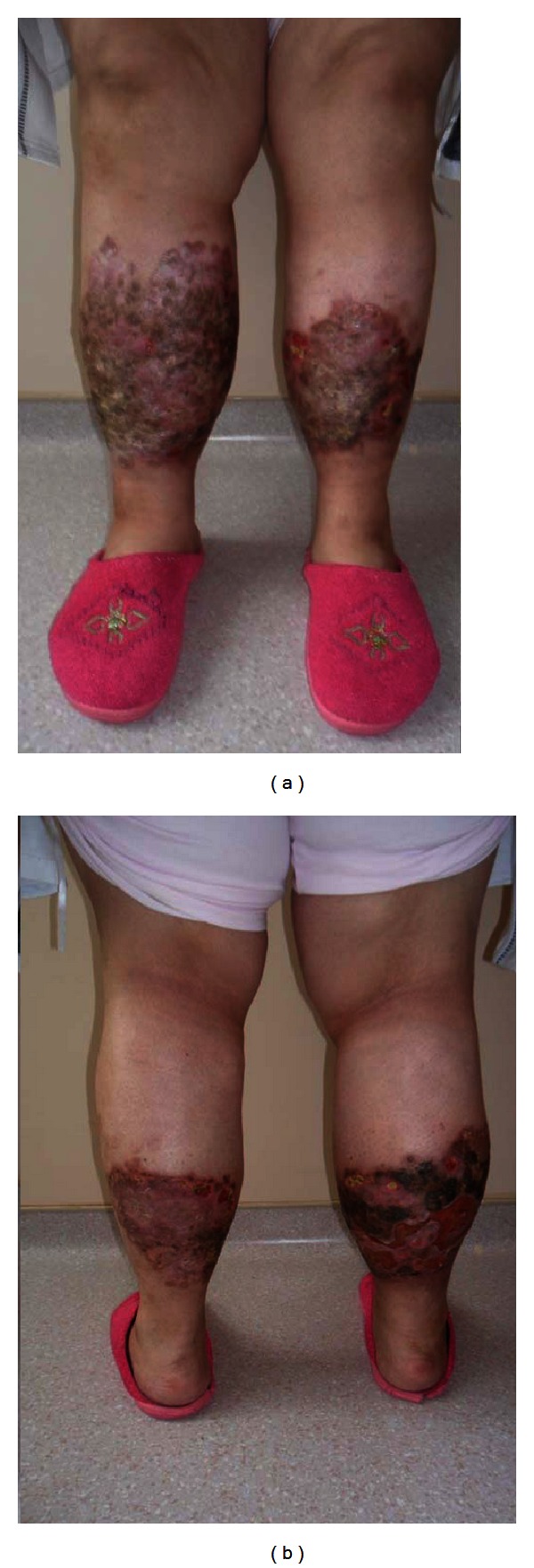
Bilateral ulcerated, oozing, and persistent plaques with depressed central areas and elevated peripheral rings over legs.

**Figure 2:**
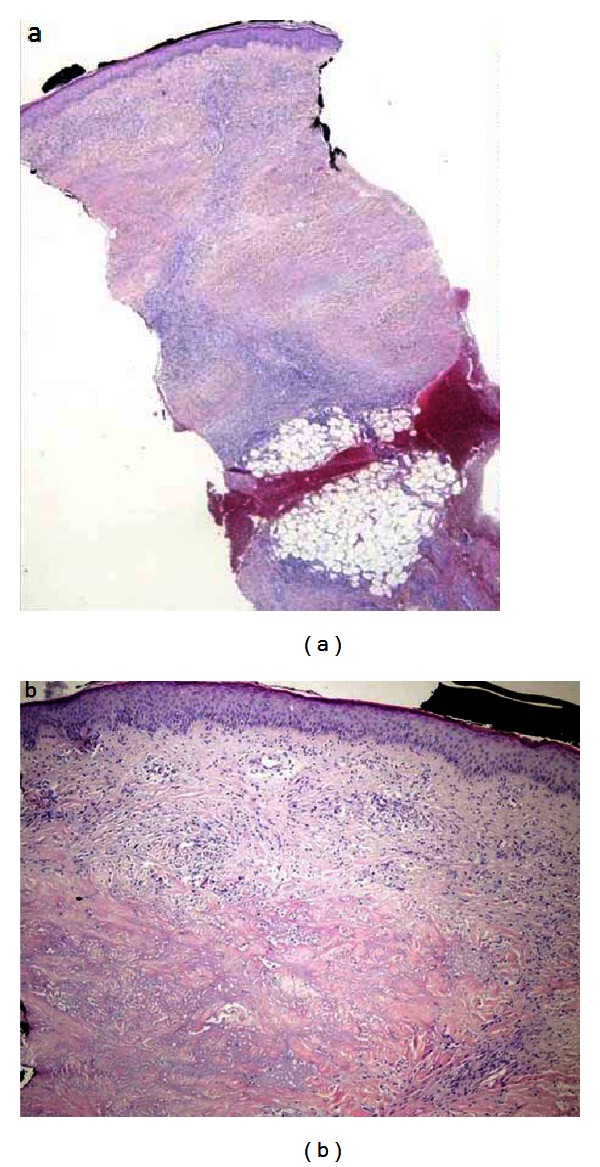
(a) The loss of epidermis rete with degenerative collagenous plaques in the dermis and peripheral histiocytic palisades (H&E ×10). (b) Subcutaneous multiple granuloma with plasma cells, lymphocytes, and multinuclear giant cells (H&E ×20).

**Figure 3:**
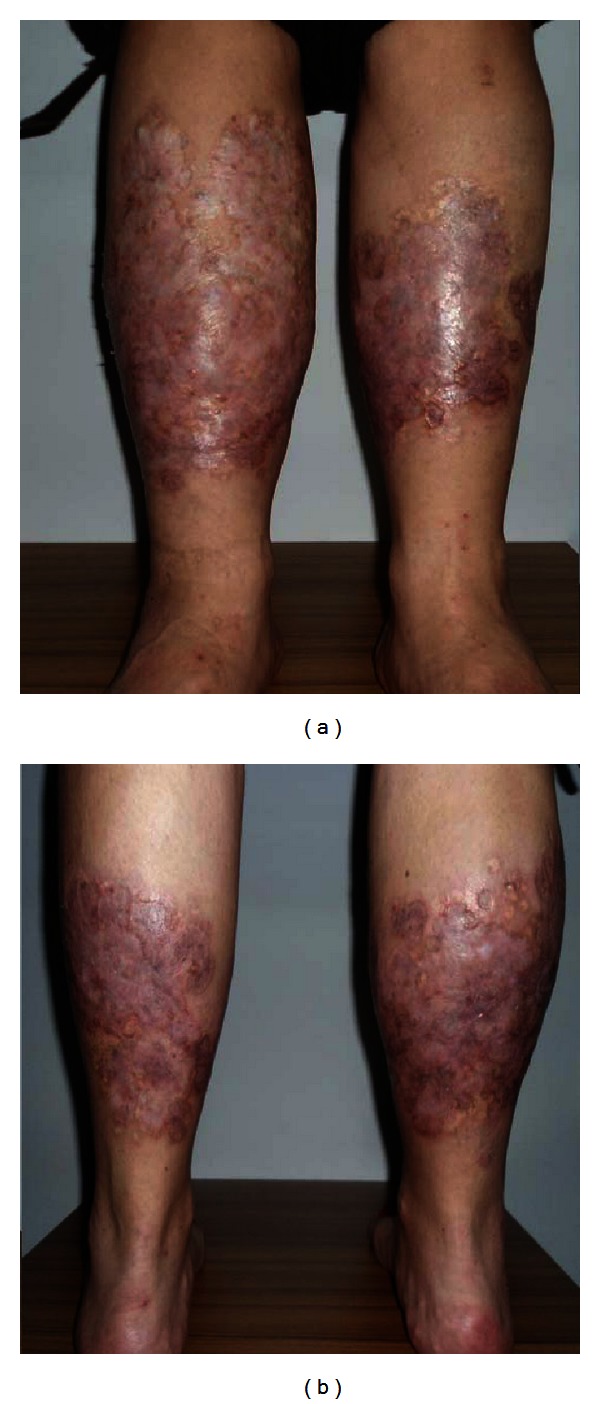
Remitted bilateral non-ulcerated and nonoozing plaques over legs after a year from the surgery.

**Figure 4:**
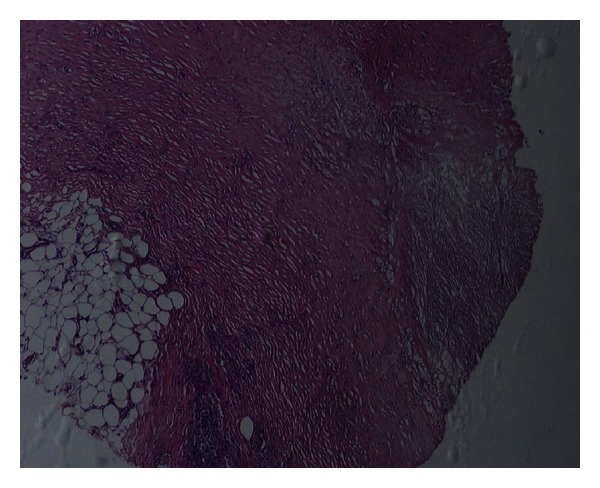
Necrotic areas with collagenous degeneration in deep dermis, peripheral fibrosis, and subcutaneous tissue panniculitis (H&E ×40).

A 32-year-old woman was operated on for morbid obesity with BMI 45,14 kg/m^2^ in April 2011. She had diabetes mellitus for 12 years. She has been under 1 U/kg/day insulin therapy since 2002 (2/3 (96 U) insulin aspart and 1/3 (30 U) insulin glargine). She had bilateral, almost circumferential, persistent plaques with depressed central areas and elevated peripheral rings over legs. The lesions are ulcerated and oozing with moderate to severe pain which partially respond to pain killers (Figure 1). The punch biopsies were performed, and the histopathologic examination of the lesions revealed the loss of epidermis rete associated with degenerative collagenous plaques in the dermis with peripheral histiocytic palisades (Figure 2(a)). Also associated subcutaneous multiple granuloma with plasma cells, lymphocytes and multinuclear giant cells (Figure 2(b)). The pathologic diagnosis was necrobiosis lipoidica. She was prescribed steroid for topical use and was treated with psoralen plus ultraviolet A photochemotherapy and also with different kind of skin dressings which were unsuccessful. She had undergone laparoscopic minigastric bypass. From the first postoperative day, she left the insulin and pain killers and after a month the lesions on the left leg subsided. She experienced the same regression of the lesions on her right leg within 3 months after surgery. She is now in her first year of her surgery with BMI 22,23 kg/m^2^ with only nonulcerating scars on her legs (Figure 3). The repeated punch biopsies revealed necrotic areas with collagenous degeneration in deep dermis, peripheral fibrosis, and subcutaneous tissue panniculitis (Figure 4).

## 3. Discussion

Modern societies are seen as environments promoting obesity, meaning that, they lead to overconsumption of food and to widespread sedentary lifestyles, which increase the risk of obesity. Excess body weight poses one of the most serious public health challenges of the 21st century for the world. According to the International Obesity Task Force and WHO, more than 1.7 billion people worldwide are overweight and more than 300 million of them are obese [1].

Obesity is strongly associated with adverse psychosocial and health consequences. T2DM and insulin resistance are the most prominent ones. From several large prospective studies [2], overweight and obesity have been estimated to account for about 65%–80% of new cases of T2DM [3]. The risk to develop DM related complications is a function of the age of onset and the duration of obesity and weight gain rate.

The resolution or improvement of T2DM after bariatric procedures has been emphasized in many studies [4] and now named and popularized as “metabolic surgery.” The explanation of such a positive result remains unclear because it is not solely correlated to weight loss. This observation suggests that the intestine plays a part in the pathogenesis of T2DM also called “enteroinsular axis” [5]. Although the specific mechanism of action underlying the early consequence of metabolic surgery has yet to be elucidated, at present at least, it appears that the major players are probably incretins, anti-incretins, and intestinal gluconeogenesis [6].

In obesity, metabolic surgery dramatically improves diabetes, often resulting in normalization of blood glucose levels and discontinuation of antidiabetic agents. In a meta-analysis and review of the literature, resolution of the clinical manifestations of DM occurred in 78.1% of patients, while DM control improved in 86.8% of the cases [7]. In their recent report, Dixon et al. documented better glycemic control after bariatric operations such as gastric bypass and sleeve gastrectomy compared to intensive medical therapy for patients with obese diabetes [8]. A randomized controlled clinical trial with consistent findings was reported by Schauer et al. [9]. They reported a significant glycemic control (glycated hemoglobin level of 6.0% or less) with remarkable weight loss after bariatric surgery. Also the use of drugs to lower glucose, lipid, and blood-pressure levels decreased significantly and the index for homeostasis model assessment of insulin resistance (HOMA-IR) improved significantly after surgery.

Necrobiosis lipoidica is a skin disease, associated mainly with DM, which is characterized by single or multiple red-brown atrophic, granulomatous plaques that are mostly located over the ventral surface of the lower legs. The course of the disease is extremely chronic, and longstanding plaques tend to ulcerate [10]. Ulceration may result in local dysesthesia, pain, infection, or cosmetic impairment. The etiology of NLD is still unknown. Microangiopathy, immune complex vasculitis with involvement of T cells, release of proinflammatory cytokines, and abnormal collagen production have all been implicated [11]. Several different therapies have been reported with limited success in the literature, including topical and systemic corticosteroids, nicotinamide, pentoxifylline, tretinoin, anti-TNF-*α*, mycophenolate mofetil, thalidomide, cyclosporine, tacrolimus, dipyridamole-aspirin, antimalarials, and PUVA [12–14]. 

In our case, we detected symptomatic, morphologic, and histopathologic remission of NLD, after bariatric surgery. The intent of surgery was primarily bariatric, but as a consequence of T2DM remission, NLD also remitted. In our knowledge this is the first reported case of NLD in the literature that remitted with bariatric/metabolic surgery. 

We suggest, therefore, that bariatric/metabolic surgery may be a promising therapeutic option for morbidly obese patients with NLD who were unresponsive to medical treatments.
